# Low Prevalence of Coronary Artery Calcium in High Cardiometabolic Risk Kenyan Adults with and Without HIV: The ASANTE Study

**DOI:** 10.5334/gh.1495

**Published:** 2025-12-05

**Authors:** Harini Shah, Srikanth Krishnan, Aditya Narashim, Sidney Korir, Geoffrey Omondi, Boni Maxime Ale, Bernard M. Gitura, John Kinuthia, Carey Farquhar, Priscilla Y. Hsue, Matthew Budoff, Chris T. Longenecker, Alfred Osoti, Saate S. Shakil

**Affiliations:** 1 Department of Medicine, University of California Los Angeles, United States; 2 Lundquist Institute at Harbor-UCLA Medical Center, Torrance, California, United States; 3 University of California Los Angeles, United States; 4 Kenyatta National Hospital, Nairobi, Kenya; 5 University of Nairobi, Nairobi, Kenya; 6 Department of Global Health, University of Washington, Seattle, United States; 7 Department of Medicine, University of Washington, Seattle, United States

**Keywords:** atherosclerosis, coronary calcium, HIV, global cardiovascular health, sub-Saharan Africa

HIV is an independent risk factor for atherosclerotic cardiovascular disease (ASCVD) despite viral suppression and adjustment for traditional risk factors. However, these data are derived largely from high-income country populations. Emerging data from East Africa, which suffers from a dual burden of HIV and a rising prevalence of cardiometabolic risk factors, indicate that coronary atherosclerosis measured by coronary artery calcium (CAC) is relatively rare in this population (1,2). We evaluated CAC among Kenyan adults with and without HIV at high cardiovascular risk.

Early Structural Cardiovascular Disease, HIV, and Tuberculosis in East Africa (ASANTE) is an ongoing cross-sectional study evaluating coronary atherosclerosis in persons with chronically treated/suppressed HIV (PWH) and HIV-uninfected controls, whose methods have been described previously (3). The study was approved by the Kenyatta National Hospital-University of Nairobi Ethical Review Committee and the University of Washington Institutional Review Board. Eligible individuals aged ≥45 years with ≥1 cardiometabolic risk factor (hypertension, diabetes, dyslipidemia, smoking, overweight/obesity), and PWH on stable antiretroviral therapy for >6 months, are recruited from Kenyatta National Hospital, Nairobi, Kenya, targeting a sample of 400 (50% female, 50% PWH). Pregnant individuals or individuals with a history of cardiovascular disease are excluded. Participants undergo non-contrast ECG-gated cardiac CT (128-slice Siemens SOMATOM Definition, Munich, Germany); CAC is quantified using the Agatston method. We present interim demographic, clinical, and CAC results stratified by HIV status. We also compare the prevalence of CAC >0 in ASANTE with other published cohorts, stratified by reported 10-year ASCVD risk (Pooled Cohort Equations [PCEs]) or, when risk was not reported, by risk estimated from other cohort characteristics (e.g., mean age). A significance threshold of <0.05 was used for *p*-values derived using two-sample *t*-tests, Fisher’s exact tests, and Chi-squared tests for continuous, binary, and categorical variables, respectively.

Table 1 summarizes demographic and clinical characteristics for 63 PWH and 42 control participants. There was no significant difference in demographics by HIV status (mean overall age 57 ± 7.2 [SD] years; 52% female). Median time since HIV diagnosis was 15.8 years. Mean 10-year ASCVD risk by PCE was higher in controls than in PWH (15.8% vs. 9.7%; *p* = 0.04); diabetes was also more prevalent in controls (64% vs. 21%; *p* < 0.0001). There was no significant difference between PWH and controls, respectively, in prevalence of hypertension (65% vs. 69%), dyslipidemia (40% vs. 57%), smoking (13% vs. 5%), overweight/obesity (73% vs. 76%), or abdominal obesity (92% vs. 81%). Mean systolic blood pressure (139 ± 20 vs. 142 ± 25 mmHg) and LDL-C (109 ± 37 vs. 95 ± 45 mg/dL) did not differ by HIV status; however, HDL-C was lower in PWH than in controls (42 ± 10 vs 52 ± 26 mg/dL; *p* = 0.03).

**Table 1:** Demographic, clinical characteristics, and cardiac CT findings in the Early Structural Cardiovascular Disease, HIV, and Tuberculosis in East Africa (ASANTE) study. *p*-values for comparisons between persons with HIV (PWH) and HIV-uninfected control participants are derived using two-sample *t*-tests (means) or Mann–Whitney–Wilcoxon tests (medians), Fisher’s exact tests, and Chi-squared tests for continuous, binary, and categorical variables, respectively.


	OVERALL *N* = 105	PWH *N* = 63	CONTROL *N* = 42	*p* (PWH vs. CONTROL)

**DEMOGRAPHIC & CLINICAL CHARACTERISTICS**				

Age, years (mean ± SD)	57 ± 7.2	56.1 ± 6.4	59 ± 8.1	0.092

Female sex, *n* (%)	55 (52)	36 (57)	19 (45)	0.24

Mean 10-year ASCVD risk score, %^§^	11.8%	9.7%	15.8%	0.040*

ASCVD risk group, *n* (%)				0.13

Low (<5%)	30 (29)	21 (33)	9 (21)	

Borderline (5–7.5%)	14 (13)	12 (19)	2 (5)	

Intermediate (7.5–10%)	15 (14)	10 (16)	5 (12)	

High (>10%)	33 (31)	17 (27)	16 (38)	

Uncategorized (predictor values out of range)	13 (12)	3 (5)	10 (24)	

Past medical history, *n* (%)				

Diabetes	40 (38)	13 (21)	27 (64)	<0.0001*

Hypertension	70 (67)	41 (65)	29 (69)	0.83

On antihypertensive treatment	61 (58)	34 (54)	27 (64)	0.32

Dyslipidemia	49 (47)	25 (40)	24 (57)	0.07

On statin therapy	11 (10)	2 (3)	9 (21)	0.007*

Current smoker	10 (10)	8 (13)	2 (5)	0.31

Time since HIV diagnosis, years (median [1^st^, 3^rd^ quartile])	—	15.8 (11, 20)	—	—

Body mass index, *n* (%)				0.67

Normal	24 (23)	16 (25)	8 (19)	

Overweight	34 (32)	21 (33)	13 (31)	

Obese	44 (42)	25 (40)	19 (45)	

Underweight	3 (3)	1 (2)	2 (5)	

Waist-to-hip ratio (mean ± SD)	0.93 ± 0.06	0.93 ± 0.06	0.93 ± 0.06	0.48

Abdominal obesity, *n* (%)^†^	92 (89)	58 (92)	34 (81)	0.13

Systolic blood pressure, mmHg (mean ± SD)	140 ± 22	139 ± 20	142 ± 25	0.42

Diastolic blood pressure, mmHg (mean ± SD)	85 ± 13	86 ± 12	84 ± 14	0.58

Total cholesterol, mg/dL (mean ± SD)	183 ± 45	186 ± 35	177 ± 55	0.35

Low-density lipoprotein cholesterol, mg/dL (mean ± SD)	103 ± 41	109 ± 37	95 ± 45	0.10

High-density lipoprotein cholesterol, mg/dL (mean ± SD)	46 ± 19	42 ± 10	52 ± 26	0.031*

eGFR, mL/min/1.73 m^2^ (mean ± SD)	79 ± 17	78 ± 16	81 ± 18	0.34

**NON-CONTRAST CARDIAC CT OUTCOMES**				

Coronary artery calcium Agatston score >0, *n* (%)	19 (18)	10 (10)	9 (21)	0.61

Agatston score (mean ± SD)	6.6 ± 23	2.9 ± 12	12 ± 33	0.086

Agatston score (median [1^st^, 3^rd^ quartile])	0 (0, 0)	0 (0, 0)	0 (0, 0)	0.34


^§^ACC/AHA Pooled Cohort Equations. ^†^Abdominal obesity defined as waist-to-hip ratio ≥0.85 for females and ≥0.9 for males.

Figure 1 A illustrates CAC by age and HIV status. CAC >0 was present in 18% of ASANTE participants, with no significant difference between PWH and controls (10% vs. 21%, *p* = 0.61; mean CAC score 2.9 ± 12 vs. 12 ± 33, *p* = 0.09). Compared to lower-risk USA-based cohorts, CAC prevalence in ASANTE was significantly lower than in the CAC Consortium of adults aged 30–50 years (34%; *p* < 0.001) and the Southern California (SoCal) cohort aged >50 years (67%; *p* < 0.0001) (4,5). CAC prevalence in ASANTE was higher than in two Ugandan studies: the Ugandan Study of HIV Effects on the Myocardium and Atherosclerosis (mUTIMA, 9%; *p* = 0.03) of intermediate-risk urban/peri-urban adults, and the Epidemiology of Coronary Artery Disease among People with HIV in Uganda Study (CAD) of borderline-risk rural adults (5%; *p* < 0.0001) (1,2). CAC in ASANTE was comparable to the Tsimane, a low-risk indigenous population in the Bolivian Amazon (15%, *p* = 0.5) (6). Figure 1B compares CAC prevalence across published cohorts.

**Figure 1:**
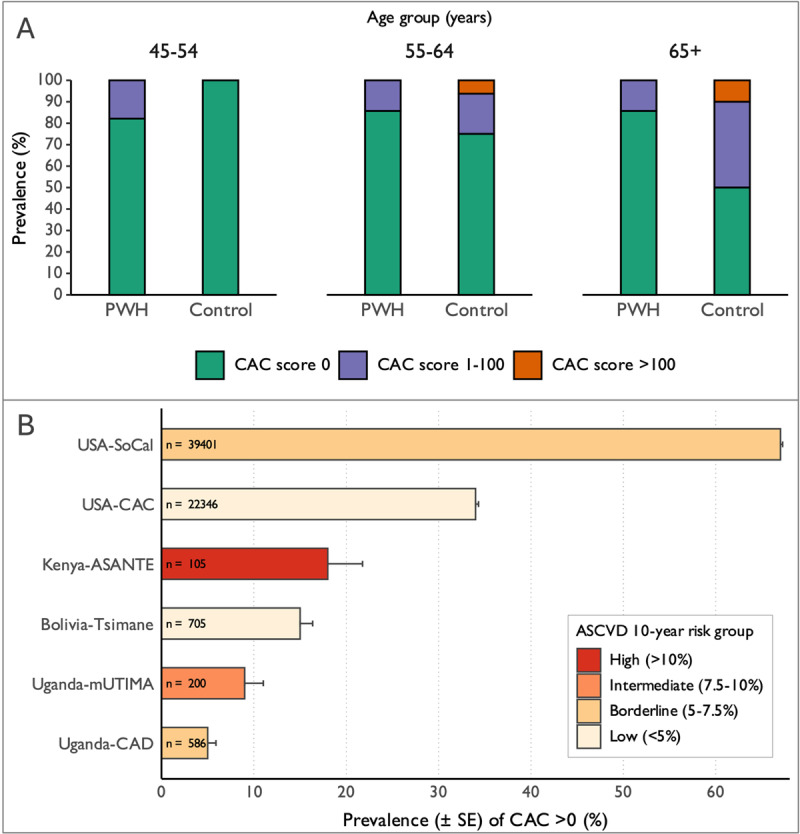
CAC stratified by age, HIV status, and geographic location. **(A)** Prevalence of coronary artery calcium (CAC) among persons with HIV (PWH) and HIV-uninfected control participants, stratified by age, in the Early Structural Cardiovascular Disease, HIV, and Tuberculosis in East Africa (ASANTE) study in Kenya. **(B)** Sample sizes and prevalence of coronary artery calcium (CAC) score >0, stratified by mean estimated 10-year ASCVD risk (ACC/AHA Pooled Cohort Equations) across published cohorts.

While limited by its cross-sectional design, modest sample size, and use of the outmoded PCEs to compare ASCVD risk with historical cohorts, our preliminary descriptive analyses highlight a striking observation: despite enriching for HIV and cardiometabolic risk factors and thus having high estimated cardiovascular risk, CAC prevalence in ASANTE was significantly lower than that observed in lower-risk general population cohorts in the United States.

Current consensus holds that HIV independently augments atherosclerotic risk based on large prospective studies in high-income regions (7). Our finding that CAC did not differ by HIV status is consistent with other high-income country studies, in which HIV is associated with increased non-calcified, but not calcified, coronary plaque compared with controls (8). This highlights a key limitation of our current study, as CAC scans cannot detect non-calcified plaque. Contrast-enhanced coronary CT angiograms in ASANTE will allow evaluation of plaque composition in future analyses.

Nevertheless, a meta-analysis of >10,000 patients in mostly high-income regions estimated a pooled CAC prevalence of 45% among PWH, compared with 10% in ASANTE. This dovetails with our key finding of paradoxically low CAC in East Africa compared with lower-risk USA-based cohorts. Several possibilities may contribute to this geographic divergence in coronary atherosclerosis epidemiology. Genetic or epigenetic factors involved in vascular plaque and calcification may vary by ancestry and geography; furthermore, endemic exposures in East Africa may modulate atherogenic pathways despite the presence of cardiometabolic comorbidities.

The role for potential unique endemic exposures and gene–environment interactions mediated via epigenetic modifications is further supported by directly comparing CAC prevalence in ASANTE (18%) with the Bolivian Tsimane (15%), an indigenous population with a subsistence lifestyle comprised of high physical activity levels and markedly low risk factor burden (e.g., <1% diabetic, 5% hypertensive, 6% obese) (6). The commensurate CAC burden between the two populations is even more striking in this context. Taken together with the extremely low CAC prevalence in urban/peri-urban and rural Uganda (9% in mUTIMA and 5% in CAD, respectively) (1,2), these observations suggest that the East African “exposome” may indeed have a mechanistically distinct effect on atherosclerosis.

In summary, our preliminary results suggest that despite enriching for cardiovascular risk factors, CAC is significantly less common in the East African population, regardless of HIV status, compared with lower-risk high-income country populations. If these hypothesis-generating findings are validated with statistical inference in a larger sample, they merit further study to identify novel atheroprotective mechanisms and exposures in this region.

## Figures and Tables

**Table:** 

ASANTE	Early Structural Cardiovascular Disease, HIV, and Tuberculosis in East Africa
ASCVD	Atherosclerotic cardiovascular disease
CAC	Coronary artery calcium
CAD	Epidemiology of Coronary Artery Disease among People with HIV in Uganda Study
HDL-C	High-density lipoprotein cholesterol
LDL-C	Low-density lipoprotein cholesterol
mUTIMA	Ugandan Study of HIV Effects on the Myocardium and Atherosclerosis
PCEs	Pooled Cohort Equations
PWH	Persons with HIV
SoCal	Southern California
USA	United States of America
